# Prevalence and Prognostic Importance of High Fibrosis-4 Index in COVID-19 Patients

**DOI:** 10.1155/2022/1734896

**Published:** 2022-05-04

**Authors:** Nurhan Demir, Bilgehan Yüzbasıoglu, Turan Calhan, Savas Ozturk

**Affiliations:** ^1^Department of Gastroenterology, Haseki Training and Research Hospital, Istanbul, Turkey; ^2^Department of Internal Medicine, Division of Nephrology, Istanbul School of Medicine, Istanbul University, Istanbul, Turkey; ^3^Department of Nephrology, Haseki Training and Research Hospital, Istanbul, Turkey

## Abstract

**Introduction:**

The fibrosis 4 (FIB-4) index was developed to predict advanced fibrosis in patients with liver disease. We aimed to evaluate the association of FIB-4 with risk factors for progression to critical illness in middle-aged patients hospitalized for coronavirus disease 2019 (COVID-19).

**Method:**

We included patients aged 35–65 years who were hospitalized following a positive RT-PCR SARS-Cov-2 test in a tertiary hospital. All data were obtained from the medical records of the patients during the first admission to the hospital. The FIB-4 index was calculated according to the equation (age (years) x AST (IU/L)/platelet count (10^9^/L)/√ALT (IU/L)). The FIB-4 index was divided into three categories according to the score categorisation: <1.3 = low risk, 1.3–2.67 = moderate risk, and >2.67 = high risk.

**Results:**

A total of 619 confirmed COVID-19 patients (mean age = 52 yrs.) were included in this study; 37 (6.0%) were admitted to the intensive care unit (ICU), of which 44% were intubated and eight (1.3%) patients died during follow-up. The results of patients with high FIB-4 scores were compared with those with low FIB-4 scores. In patients with high FIB-4 scores, male gender, and advanced age, decreased neutrophil, lymphocyte, thrombocyte, and albumin counts, elevated AST, LDH, CK, ferritin, CRP, and D-dimer, and low GFR were the high-risk factors for critical illness. Additionally, the number of patients referred to ICU with high FIB-4 who died had higher scores than from those with low scores.

**Conclusion:**

The FIB-4 index derived from baseline data obtained during hospitalisation can be used as a simple, inexpensive, and straightforward indicator to predict ICU requirement and/or death in middle-aged hospitalized COVID-19 patients.

## 1. Introduction

Patients with acute respiratory syndrome caused by the SARS-COV-2 infection (COVID-19) present a wide range of disease severity on a spectrum from asymptomatic illness to needing treatment at the intensive care unit (ICU): and organ support. A variety of clinical and laboratory findings have been identified on admission. In studies where COVID-19 patients' initial characteristics, clinical symptoms, laboratory parameters, and other risk factors for adverse outcomes have been analysed, high C-reactive protein (CRP) and D-dimer and low platelet counts, fibrinogen, lymphocyte, and albumin levels were associated with severe COVID-19 [1, 2].

SARS-CoV-2 binds to the ACE2 receptors on target cells via the receptor-binding domain (RBD) on the viral particle that enters the cell and leads to replication. ACE2 receptors are expressed in the gastrointestinal tract, vascular endothelium, and cholangiocytes of the liver [3, 4]. Liver damage may be associated with the direct cytopathic effect of the virus, excessive immune reaction, sepsis, or medication. It has been shown that patients with abnormal liver function tests (LFTs) have stayed longer in hospital [5] and the aspartate and alanine transaminase (AST and ALT, respectively) levels are higher in severe COVID-19 patients [6]. Usually, AST levels are higher than ALT levels in severe COVID-19 patients [6].

In COVID-19 disease, when higher AST elevation versus ALT, also creatine kinase (CK) and lactate dehydrogenase (LDH) elevations, is taken into consideration, it becomes difficult to distinguish whether the liver test elevations are due to the SARS-CoV-2 infection itself or other reasons. Some of the possible factors are muscle damage secondary to extrahepatic events, cytokine release syndrome, ischemia, and drug-induced liver injury [7, 8].

The fibrosis 4 (FIB-4) index was developed to predict advanced fibrosis in patients with liver disease. It is a predictor of mortality in patients admitted to the ICU [9] and is associated with adverse outcomes, including the need for invasive mechanical ventilation and death [10]. It has also been shown that this index is higher in patients followed up for severe COVID-19-related pneumonia. A meta-analysis has shown it to be associated with mortality [11], while in another study, the authors suggested that it was associated with mortality independently of underlying reasons, including liver disease [9]. Additionally, while increasing FIB-4 index levels were associated with poor clinical outcomes, a FIB-4 score of over 2.67 has been suggested as an independent risk factor in COVİD-19 patients [12, 13].

The FIB-4 index calculation is based on simple blood tests using age, AST and ALT levels, and platelet counts [14]. Considering that AST is higher than ALT in the inflammatory response and that the age of the patient is used as a factor in the calculation, the FIB-4 index may also be used as a marker to indicate a poor COVID-19 prognosis. In the evaluation made by adding the FIB-4 index to the model employing traditional cardiovascular risk factors, liver fibrosis has been found to be associated with an increased risk of all-cause mortality and cardiovascular mortality [15]. It has also been found that the burden of liver fibrosis, but not steatosis, is an independent predictor of all-cause and cardiovascular mortality during the long-term follow-up of patients with ischemic stroke (IS) [13].

FIB-4 index scores have similar accuracy for advanced fibrosis in patients over 35 years of age. However, the specificity for advanced fibrosis is unacceptably low in patients aged 65 and higher, resulting in a high false-positive rate [16]. Specifically, *a* FIB-4 score of <1.30 is accepted as low risk for fibrosis, and a FIB-4 score of >2.67 is accepted as high risk. Some studies have indicated that the FIB-4 index might play an important role in the prognosis of the disease course of COVID-19. In a number of these studies, the FIB-4 index has been related to liver fibrosis caused by metabolic-associated liver disease (MAFLD), which occurs at rates as high as 2.8–5.6% of the general population and 18% in high-risk groups like type 2 diabetes mellitus (T2DM) [17–20]. However, it was not possible to screen these patients for liver steatosis with imaging methods during the disease. In later studies, it was shown that the relationship between the FIB-4 index and increased mortality in patients with a COVID-19 diagnosis was not associated with metabolic disease [21].

In this study, we aimed to evaluate the association of FIB-4 with risk factors for progression to critical illness in middle-aged patients hospitalized for COVID-19.

## 2. Methods

This retrospective study cohort followed the report “Strengthening the Reporting of Observational Studies in Epidemiology” (STROBE) [22]. It was approved by the Ethics Committee of the Health Sciences University Haseki Training and Research Hospital (no. 2020-44).

### 2.1. Study Design and Participants

This study included 619 of 1165 selected hospitalized patients with SARS-CoV-2 infection in a tertiary hospital in the Istanbul region between April 21, 2020, and June 30, 2020, where follow-up was terminated by hospital discharge or death. The diagnosis was made by real-time reverse transcriptase-polymerase chain reaction (rRT-PCR) detection of SARS-CoV-2 RNA in a nasopharyngeal swab sample. Patients diagnosed with other illnesses, solid organ transplant recipients, and patients treated with drugs known to produce myelotoxicity were excluded from the study, along with patients with SARS-CoV-2 infection but hospitalized for reasons other than COVID-19.

### 2.2. Definition

All cases of COVID-19 were diagnosed according to the WHO interim guidelines with the whole SARS-CoV-2 sequence being PCR-positive. Fever was defined as a tympanic temperature of 37.5°C or higher. Systemic inflammatory response syndrome (SIRS) at presentation was defined as meeting any two of the following criteria: (i) white blood cell count (WBC) < 4000 cells/mm or >12000 cells/mm, (ii) body temperature <36°C or >38°C, (iii) heart rate >90 beats/min, and (iv) tachypnea >20 breaths/min. Persistent hypotension was defined as mean systemic arterial pressure (MAP) < 65 mmHg, despite volume resuscitation and vasopressors.

Acute respiratory distress syndrome (ARDS) was defined in accordance with the WHO interim guidelines. Acute kidney injury was defined by peak serum creatinine (>0.3 mg/dL within 48 hours or 1.5 × baselines within 7 days) and/or decreased urine output (<0.6 mL/kg/hour for 6 hours) at the time of admission. According to oxygen demand, two groups were defined as follows: a low-dose oxygen group, using respiratory support, nasal cannula, or venturi mask, and a high-dose oxygen group, using a high-flow nasal cannula, invasive mechanical ventilation, and/or extracorporeal membrane oxygenation (ECMO).

### 2.3. Noninvasive Evaluation of Liver Fibrosis

The FIB-4 index was calculated according to the following equation: age (years) x AST (IU/L)/[platelet count (10^9^/L)/√ALT (IU/L)] [14].

The FIB-4 score was calculated from blood tests performed at the time of admission before initiating any specific COVID-19 therapy [16]. A FIB-4 score of below 1.30 was taken to show a low risk of advanced fibrosis, a FIB-4 score of above 2.67 was regarded as high risk, and scores between 1.30 and 2.67 were considered moderate. The study patients were further divided as FIB-4 risk group using the previously published breakpoints.

### 2.4. Statistical Analysis

The descriptive statistics were presented as numbers, categorical variables are presented as percentages, and the mean, standard deviation, minimum value, maximum value, and median are presented as numerical variables. Comparisons of numerical variables in two independent groups were made using the Student's *t*-test when the normal distribution condition was met and the Mann–Whitney *U* test when it was not met. The ratios in the groups were analysed using the Chi-square test. The statistical alpha significance level was accepted as *p* < 0.05. IBM SPSS Statistics version 26 for Windows (IBM Corp., Armonk, NY, USA) was used for statistical analyses.

## 3. Results

### 3.1. Demographic and Baseline Characteristics

A total of 1165 patients hospitalized and followed up in the clinic with SARS-CoV-2 infection and confirmed by a positive rRT-PCR test between April 21, 2020, and June 30, 2020, were evaluated. Of these, 619 people met the study inclusion criteria.

The baseline characteristics of the total (*n* = 619) study population are given in Table 1. The median follow-up period of the patients was eight days. A total of 37 (6.0%) of the 619 patients needed ICU, and 15/37 (44%) patients were intubated in the ICU, while eight (1.3%) patients died during follow-up. The mean age of our patients was 52 years (IQR, 45–58 years), and there was a higher predominance of males (58.6%). The most common comorbidity in the patients was hypertension, diagnosed in 206 (33.3%) patients. The other comorbidities listed in order of frequency by patient number (percentage) were as follows: history of diabetes mellitus 154 (24.7%), ischemic heart disease 39 (6.4%), chronic obstructive pulmonary disease (COPD) 27 (4.4%), collagenous/autoimmune disease 25 (4.1%), chronic kidney disease 16 (2.6%), and heart failure and active malignancy 12 (2.0%) (Table 2).

The diagnostic workup data (baseline clinical findings and laboratory blood parameters) of 42 (6.8%) patients who were admitted to the ICU and/or died (COVID-19 related) were reevaluated for prominent risk factors. In male patients (76.2%), ischemic heart disease, chronic kidney disease, and cerebrovascular disease, low lymphocyte count, low albumin, high LDH, high ferritin, high C-reactive protein (CRP), high procalcitonin, and high D-dimer were considered as the high-risk factors for critical illness (Table 2).

### 3.2. Main Features by FIB-4 Categories

The diagnostic workup data during the first admission to the hospital were evaluated with the basic clinical features and laboratory test results according to the FIB-4 categories (Table 1).

#### 3.2.1. Results of Patients with FIB 4 > 2.67

**Figure 1:**
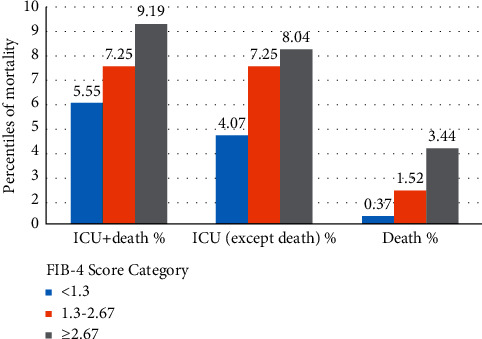
The number of patients admitted to ICU and their death rates according to the FIB-4 score. FIB-4, fibrosis index; ICU, intensive care unit.

In patients with FIB-4 >2.67, male gender (70.1%), advanced age (median [IQR] 55, *p* < 0.001), neutropenia (IQR; 2830, *p* < 0.001), lymphopenia (IQR: 1090, *p* < 0.001), thrombocytopenia (IQR: 120, *p* < 0.001), low albumin (IQR: 3.6, *p* < 0.001), high AST (IQR: 50, *p* < 0.001), high creatinine (IQR: 0.865 [mg/dL], *p* < 0.001), high LDH (IQR:398 [Iu/L], *p* < 0.001), high CK (IQR:366, *p* < 0.001), high ferritin (IQR: 330.1 (ng/ml), *p* < 0.001), elevated CRP (95.4%, *p* < 0.001), D-dimer elevation (53.5%, *p* < 0.001), and low GFR (IQR: 98.7, *p* < 0.001) were found to be statistically high (Figure 1).

## 4. Discussion

In this study, the FIB-4 score derived from routine baseline laboratory values of hospitalized COVID-19 patients has been found to be significantly associated with clinical outcomes associated with COVID-19. SARS-CoV-2-related inflammation and potentially direct virological effects presumably mediate this association. We think that laboratory data indicating a poor prognosis for the patient, skeletal muscle damage, SARS-CoV-2 infection, and hepatocellular and portal system changes due to systemic inflammation all play important roles and possibly cause multifactorial elevation of FIB-4. For this reason, the increase in FIB-4 levels may be related to the pathogenesis of COVID-19 and can thus function as an indicator of a severe course of the disease.

In this study, we observed the factors affecting the severe course of the disease in middle-aged patients. Along with previous studies [1, 2, 9, 10], we found high fever, dyspnoea, anorexia, fatigue, high levels of LDH, ferritin, D dimer, procalcitonin, and CRP, and low levels of thrombocyte, lymphocyte, and albumin at first hospital admission to be poor prognostic factors for mortality/ICU admission. In addition, our study supported findings that ischemic heart diseases, chronic kidney diseases, cerebrovascular diseases, and comorbidities were poor prognostic factors related to mortality/ICU admission, as previously reported in CDC COVİD-19 data [23].

Considering that the FIB-4 score can be calculated as high due to the liver enzyme elevation secondary to COVID-19 infection and/or the drugs used, we calculated the FIB-4 score using the recorded results of the patients' first admission to the health institution (immediately after the positive SARS-CoV-2 RT-PCR result) in order to avoid misleading results. Since fibrosis values have been reported as low in patients under 35 years of age and exaggeratedly high in patients over 65 [24], only patients aged 35–65 years were included in this study.

FIB-4 components are not specific to the liver and may be affected by disorders other than liver disease. To protect our test results from these factors, patients with previously known muscle or liver diseases, receiving chemotherapy, and with blood disorders that may cause thrombocytopenia or liver enzyme disorders were excluded from the study. Abnormalities in liver enzymes are common and associated with the severity and prognosis of COVID-19 [25]. Predominantly elevated AST levels reflect the severity of disease and actual liver damage in COVID-19 [26]. Contrarily, Liv et al. reported their concerns about the interpretation of aspartate aminotransferase and reported that AST-based liver damage could be overestimated in COVID-19 patients [27]. In recent studies, elevated AST and ALT levels have been reported in severe COVID-19 disease with end-organ damage [5]. Liver enzymes and FIB-4 index increase independently of underlying liver diseases in patients with COVİD-19 [28]. The FIB-4 index has been found to be a useful predictive marker for mortality in patients with COVID-19 regardless of its severity [29].

Here, FIB-4 index scores were categorized as follows: <1.3 = low risk, 1.3–2.67 = medium risk, and >2.67 = high risk. According to our study results, patients with high-risk FIB-4 scores were predominantly male, of advanced age, with neutropenia, lymphopenia, low albumin, thrombocytopenia, and GFR, and high AST, creatinine, LDH, CK, ferritin, CRP, procalcitonin, and D-dimer. The association between FIB-4 and the risk of progression to critical illness in middle-aged patients with COVID-19 has been reported in another study supporting the present one. Therefore, the risk of advanced fibrosis was estimated in 28.1% of patients, and patients with a FIB-4 score of ≥2.67 more frequently required mechanical ventilation [13].

The FIB-4 index, which was designed for the assessment of liver fibrosis, has also been shown to be a useful risk score in nonhepatic diseases, such as atrial fibrillation and intracerebral haemorrhage [30]. Higher FIB-4 scores in COVID-19 patients have been associated in previous studies with higher inflammatory marker levels, increased mechanical ventilation requirement, and higher mortality [10, 20]. Also, liver fibrosis burden other than steatosis has been associated with all-cause mortality, increased risk of cardiovascular mortality, and IS recurrence in patients with high FIB-4 index [15, 31].

We compared the poor prognostic factors, as discussed in the previous studies [1, 2, 6, 23, 32, 33], in the FIB-4 ≥ 2.67 patient group and in middle-aged patients with severe course of the disease (in the mortality/ICU admission group). We found that poor prognostic factors such as advanced age, male gender, neutropenia, lymphopenia, low albumin, thrombocytopenia, and GFR and high AST, creatinine, LDH, CK, ferritin, CRP, procalcitonin, and D-dimer were common in these two groups. This clearly shows that FIB-4 works well for severe COVID-19 prognosis in this group of patients.

Our study has some limitations. It was retrospective and did not have a control group. We only included patients aged between 35 and 65 because the FIB-4 score was validated in this age group, so we do not know whether these results can be extrapolated to patients with more increased risk of mortality, such as elderly patients suffering from COVID-19. As we included middle-aged patients, mortality in our cohort was not so high. Therefore, we could not perform multivariate analyses of the relationship between FIB-4 score and mortality. During the pandemic, we sometimes hospitalized some asymptomatic mildly diseased patients (with asymptomatic or normal CT) because of high-risk factors or for isolation. This may be another reason for the low mortality in our study. This enabled the FIB-4 score to be tested in a cohort of severe-critical patients and mild-asymptomatic patients together.

We could not evaluate possible correlations between cytokine levels and FIB-4 during a pandemic. We did not have elastography and liver biopsy results to confirm the increase in FIB-4 as a result of liver stiffness or fibrosis. We did not have patient body mass index results to evaluate the effects of obesity. We excluded patients with chronic liver disease in order to prevent outliers.

In conclusion, our study has shown that the FIB-4 score calculated using laboratory data of middle-aged patients obtained during hospitalisation for COVID-19 could be a practical, easy and inexpensive indicator for estimating ICU referral and/or in-hospital mortality.

## Figures and Tables

**Table 1:** Baseline demographics, clinical findings, and laboratory test data categorized according to the FIB-4 index at diagnosis.

		Total (*n* = 619)	FIB-4 <1.3 *n* = 270 (43.6%)	FIB-4 1.3–2.67 *n* = 262 (42.3%)	FIB-4 >2.67 *n* = 87 (14.1%)	*p*
Gender *n* (%)	Male	363 (58.6)	146 (54.1)	156 (59.5)	61 (70.1)	0.028
	Female	256 (41.4)	124 (45.9)	106 (40.5)	26 (29.9)	

Age (years) median (IQR)		52 (45–58)	49 (43–55)	53 (47–59)	55 (50–61)	<0.001

Comorbidities *n* (%)	Diabetes mellitus	153 (24.7)	62 (23.0)	65 (24.8)	26 (29.9)	0.428
	Hypertension	206 (33.3)	80 (29.6)	99 (37.9)	27 (31.0)	0.113
	Ischemic heart disease	39 (6.4)	10 (3.7)	20 (7.8)	9 (10.3)	0.044
	Heart failure	12 (2.0)	2 (0.7)	8 (3.1)	2 (2.3)	0.145
	Chronic kidney disease	16 (2.6)	6 (2.2)	8 (3.1)	2 (2.3)	0.821
	COPD	27 (4.4)	9 (3.4)	16 (6.2)	2 (2.3)	0.175
	Active malignancy	12 (2.0)	0 (0.0)	7 (2.7)	5 (5.7)	<0.001
	Collagenous/autoimmune disease	25 (4.1)	14 (5.2)	8 (3.1)	3 (3.4)	0.436
	Cerebrovascular/neurological disease	11 (1.8)	4 (1.5)	5 (1.9)	2 (2.3)	0.773
	Cardio-cerebrovascular disease	49 (8.0)	14 (5.2)	24 (9.2)	11 (12.6)	0.051

Time between first symptom and diagnosis (days) median (IQR)		4.5 (3–7)	5 (3–7)	5 (3–7)	4 (2–6.75)	0.174

Symptoms on admission *n* (%)	Fever	290 (47.0)	119 (44.2)	127 (48.5)	44 (51.2)	
	Fatigue	292 (47.5)	123 (45.6)	123 (47.3)	46 (54.1)	0.386
	Shortness of breath	196 (31.7)	74 (27.4)	98 (37.4)	24 (27.6)	0.031
	Dry cough	374 (60.4)	156 (57.8)	170 (64.9)	48 (55.2)	0.137
	Cough with phlegm	41 (6.6)	15 (5.6)	20 (7.6)	6 (6.9)	0.625
	Anorexia	27 (4.4)	9 (3.3)	10 (3.8)	8 (9.2)	0.057
	Muscle pain	143 (23.1)	65 (24.1)	61 (23.3)	17 (19.5)	0.681
	Throat ache	85 (13.7)	40 (14.8)	33 (12.6)	12 (13.8)	0.758
	Headache	94 (15.2)	40 (14.8)	37 (14.1)	17 (19.5)	0.463
	Diarrhoea	50 (8.1)	24 (8.9)	19 (7.3)	7 (8.0)	0.787
	Anosmia	30 (4.8)	12 (4.4)	14 (5.3)	4 (4.6)	0.884

COVID-19-related clinical status at time of diagnosis *n* (%)	Asymptomatic	29 (4.7)	17 (6.3)	10 (3.8)	2 (2.3)	0.210
	Asymptomatic or mild disease	518 (83.7)	234 (86.7)	216 (82.4)	68 (78.2)	0.135
	Moderate-to-severe disease	101 (16.3)	36 (13.3)	46 (17.6)	19 (21.8)	

Laboratory findings at time of diagnosis median (IQR)	Haemoglobin (g/dl)	13.3 (12.1–14.3)	13.3 (12.1–14.3)	13.3 (12.3–14.3)	13.1 (11.8–14.4)	0.367
	Neutrophil count (/mm^3^)	3550 (2600–5000)	4205 (3007.5–5622.5)	3290 (2430–4832.5)	2830 (2200–3950)	<0.001
	Lymphocyte count (/mm^3^)	1360 (960–1860)	1635 (1197.5–2100)	1240 (860–1610)	1090 (770–1440)	<0.001
	Platelet count (×1000/mm^3^)	200 (155–253)	251 (206.75–307.75)	178.5 (149–215)	120 (100–145)	<0.001
	Erythrocyte sedimentation rate (mm/hr)	36 (16–59)	33 (12–54)	40 (19–61)	37.5 (18–45.5)	0.043
	Creatinine (mg/dL)	0.84 (0.7–0.99)	0.82 (0.67–0.98)	0.85 (0.7–0.99)	0.865 (0.73–1.09)	0.017
	eGFR median (IQR)	110.4 (82.8–152.4)	116.8 (90.0–159.8)	108.5 (81.7–148.6)	98.7 (66.9–138.2)	0.002
	Albumin (g/dl)	3.8 (3.4–4.1)	3.8 (3.5–4.1)	3.7 (3.4–4)	3.6 (3.35–3.9)	0.008
	AST (IU/L)	28 (22–40)	23 (18–30)	31 (24.75–42.25)	50 (34–76)	<0.001
	ALT (IU/L)	27 (19–41)	27 (18–40)	27 (19–41.25)	31 (21–56)	0.047
	LDH (IU/L)	312 (237–423.75)	285 (214.5–383)	311.5 (243–427.25)	398 (315–535)	<0.001
	CK (IU/L)	88 (57.75–175.5)	72 (52–109.5)	99 (61.5–187)	205.5 (89.75–507.25)	<0.001
	Amylase (IU/L))	59 (47–80)	57 (45–74)	58.5 (48–82)	73 (47.5–91.5)	0.062
	Lipase (IU/L)	34.7 (21.3–54.2)	31 (20–46)	35 (22.6–57.5)	39 (24.5–66)	0.046
	Ferritin (ng/ml)	200 (109–441)	166 (83–317.25)	228 (126.5–483.5)	330.1 (166.75–749.4)	<0.001
	Lymphopenia n (%)	242 (39.1)	67 (24.8)	124 (47.3)	51 (58.6)	<0.001
CRP *n* (%)	Normal	87 (14.1)	62 (23.0)	21 (8.0)	4 (4.6)	<0.001
	1–5 times increase	208 (33.6)	91 (33.7)	94 (35.9)	23 (26.4)	
	5–10 times increase	124 (20.0)	49 (18.1)	58 (22.1)	17 (19.5)	
	10–20 times increase	106 (17.1)	38 (14.1)	45 (17.2)	23 (26.4)	
	>20 times increase	94 (15.2)	30 (11.1)	44 (16.8)	20 (23.0)	

Procalcitonin at diagnosis n (%)	Normal	453 (83.9)	209 (90.1)	188 (82.1)	56 (70.9)	<0.001
	High	87 (16.1)	23 (9.9)	41 (17.9)	23 (29.1)	

D-dimer *n* (%)	Normal	339 (57.5)	172 (66.7)	127 (51.6)	40 (46.5)	<0.001
	1––<3 times	162 (27.5)	53 (20.5)	87 (35.4)	22 (25.6)	
	>3 times	89 (15.1)	33 (12.8)	32 (13.0)	24 (27.9)	

CT imaging *n* (%)		611 (98.9)	266 (98.5)	260 (99.2)	85 (98.8)	0.776

CT findings *n* (%)	Single lesion	20 (3.3)	8 (3.0)	5 (1.9)	7 (8.2)	<0.001
	Unilateral multiple lesion	43 (7.1)	30 (11.4)	11 (4.3)	2 (2.4)	
	Bilateral multiple lesion	515 (84.8)	209 (79.2)	233 (90.3)	73 (85.9)	
	Completely normal	29 (4.8)	17 (6.4)	9 (3.5)	3 (3.5)	

Diagnosis-follow-up duration days median (IQR)		8 (5–12)	7 (5–10)	8 (6–13)	11 (8–12)	<0.001
ICU admission *n* (%)		37 (6.0)	11 (4.1)	19 (7.3)	7 (8.0)	0.206
Intubation *n* (%)		15 (44.1)	6 (60.0)	5 (29.4)	4 (57.1)	0.261

ALT, alanine aminotransferase; AST, aspartate aminotransferase; CK creatinine kinase; COPD, chronic obstructive pulmonary disease; CRP, C-reactive protein; CT, computerised thorax tomography; FIB-4, fibrosis index; eGFR, estimated glomerular filtration rate; GGT,-glutamyl transpeptidase; ICU, intensive care unit; INR, international normalized rate; LDH, lactate dehydrogenase.

**Table 2:** Baseline demographics, clinical characteristics, and laboratory data of all patients at the time of diagnosis according to needing ICU referral and/or deceased.

		ICU admission and/or deceased		*p*
		Yes *n* = 42 (6.8%)	No *n* = 577 (93.2%)	
Gender *n* (%)	Male	32 (76.2)	331 (57.4)	0.017
	Female	10 (23.8)	246 (42.6)	

Age (years) median (IQR)			52 (45–58)	0.742

Comorbidities *n* (%)	Diabetes mellitus	15 (35.7)	138 (23.9)	0.087
	Hypertension	16 (38.1)	190 (33.0)	0.498
	Ischemic heart disease	7 (16.7)	32 (5.6)	0.013
	Heart failure	2 (4.8)	10 (1.8)	0.197
	Chronic kidney disease	4 (9.5)	12 (2.1)	0.019
	COPD	4 (9.5)	23 (4.0)	0.105
	Active malignancy	2 (4.8)	10 (1.7)	0.195
	Collagenous/autoimmune disease	2 (4.8)	23 (4.0)	0.685
	Cerebrovascular/neurological disease	1 (2.4)	10 (1.7)	0.543
	Cardio-cerebrovascular disease	8 (19.0)	41 (7.1)	0.013

Time between first symptom and diagnosis (days) median (IQR)			4 (3–7)	0.872

Symptoms at admission *n* (%)	Fever	27 (64.3)	263 (45.7)	0.020
	Fatigue	27 (64.3)	265 (46.2)	0.024
	Shortness of breath	19 (45.2)	177 (30.7)	0.050
	Dry cough	27 (64.3)	347 (60.1)	0.596
	Cough with phlegm	4 (9.5)	37 (6.4)	0.513
	Anorexia	7 (16.7)	20 (3.5)	0.001
	Muscle pain	12 (28.6)	131 (22.7)	0.384
	Throat ache	8 (19.0)	77 (13.3)	0.300
	Headache	7 (16.7)	87 (15.1)	0.782
	Diarrhoea	4 (9.5)	46 (8.0)	0.767
	Anosmia	4 (9.5)	26 (4.5)	0.138
COVID-19-related clinical status at the time of diagnosis *n* (%)	Asymptomatic	1 (2.4)	28 (4.9)	0.713
	Asymptomatic or mild disease	20 (47.6)	498 (86.3)	<0.001
	Moderate-to-severe illness	22 (52.4)	79 (13.7)	
Laboratory findings at the time of diagnosis median (IQR)	Haemoglobin (g/dl)	13 (10.7–14.3)	13.3 (12.2–14.3)	0.098
	Neutrophil count (/mm^3^)	4255 (3070–7142.5)	3520 (2580–4980)	0.043
	Lymphocyte c(/mm^3^)	945 (705–1377.5)	1400 (1000–1890)	<0.001
	Platelet count (×1000/mm^3^)	198.5 (150.5–276.5)	200 (156–252)	0.973
	Erythrocyte sedimentation rate (mm/hr)	38 (16–68)	36 (16–59)	0.706
	Creatinine (mg/dL)	0.92 (0.7–1.2)	0.84 (0.695–0.99)	0.158
	Albumin (g/dl)	3.4 (2.9–3.8)	3.8 (3.5–4.1)	<0.001
	AST (IU/L)	31.5 (22–53.75)	28 (22–40)	0.265
	ALT (IU/L)	26.5 (18–45.75)	27 (19–41)	0.730
	LDH (IU/L)	366 (292.25–546)	307.5 (234–420.25)	0.010
	CK (IU/L)	105 (74–263)	87 (57–174)	0.099
	Amylase (IU/L))	63 (49.5–89)	59 (47–79.5)	0.368
	Lipase (IU/L)	35 (18–55)	34.41 (21.34–54)	0.956
	Ferritin (ng/ml)	468.3 (238–824.25)	191 (105.3–404.5)	<0.001
	Lymphopenia *n* (%)	27 (64.3)	215 (37.3)	0.001

CRP *n* (%)	Normal	1 (2.4)	86 (14.9)	<0.001
	1–5 times increase	6 (14.3)	202 (35.0)	
	5–10 times increase	3 (7.1)	121 (21.0)	
	10–20 times increase	12 (28.6)	94 (16.3)	
	>20 times increase	20 (47.6)	74 (12.8)	

Procalcitonin *n* (%)	Normal	22 (57.9)	431 (85.9)	<0.001
	High	16 (42.1)	71 (14.1)	

D-dimer *n* (%)	Normal	16 (39.0)	323 (58.8)	<0.001
	1––<3 times	10 (24.4)	152 (27.7)	
	>3 times	15 (36.6)	74 (13.5)	

CT *n* (%)		40 (95.2)	571 (99.1)	0.076

CT findings *n* (%)	Single lesion	0 (0.0)	20 (3.5)	0.387
	Unilateral multiple lesion	1 (2.5)	42 (7.4)	
	Bilateral multiple lesion	36 (90.0)	479 (84.5)	
	Completely normal	3 (7.5)	26 (4.6)	

Length of hospital stay (days) median (IQR)		15 (7.75–23.25)	8 (5–12)	<0.001

ALT, alanine aminotransferase; AST, aspartate aminotransferase; CK creatinine kinase; COPD, chronic obstructive pulmonary disease; CRP, C-reactive protein; CT, computerised thorax tomography; FIB-4, fibrosis index; eGFR, estimated glomerular filtration rate; GGT, glutamyl transpeptidase; ICU, intensive care unit; INR, international normalized rate; LDH, lactate dehydrogenase; eGFR, estimated glomerular filtration rate.

## Data Availability

All data are available upon request.

## References

[1] Shi S., Nie B., Chen X. (2021). Clinical and laboratory characteristics of severe and non‐severe patients with COVID‐19: a retrospective cohort study in China. *Journal of Clinical Laboratory Analysis*.

[2] Kermali M., Khalsa R. K., Pillai K., Ismail Z., Harky A. (2020). The role of biomarkers in diagnosis of COVID-19—a systematic review. *Life Sciences*.

[3] Xu X., Chen P., Wang J. (2020). Evolution of the novel coronavirus from the ongoing Wuhan outbreak and modeling of its spike protein for risk of human transmission. *Science China Life Sciences*.

[4] Hamming I., Timens W., Bulthuis M., Lely A., Navis G., Van Goor H. (2004). Tissue distribution of ACE2 protein, the functional receptor for SARS coronavirus. A first step in understanding SARS pathogenesis. *The Journal of Pathology*.

[5] Huang C., Wang Y., Li X. (2020). Clinical features of patients infected with 2019 novel coronavirus in Wuhan, China. *The Lancet*.

[6] Cao W., Shi L., Chen L., Xu X., Wu Z. (2020). Clinical features and laboratory inspection of novel coronavirus pneumonia (COVID-19) in Xiangyang, Hubei. *MedRxiv*.

[7] Zhang C., Shi L., Wang F.-S. (2020). Liver injury in COVID-19: management and challenges. *The Lancet. Gastroenterology and Hepatology*.

[8] Hundt M. A., Deng Y., Ciarleglio M. M., Nathanson M. H., Lim J. K. (2020). Abnormal liver tests in COVID‐19: a retrospective observational cohort study of 1,827 patients in a major US hospital network. *Hepatology*.

[9] Li Y., Regan J., Fajnzylber J. (2020). Liver fibrosis index FIB-4 is associated with mortality in COVID-19. *Hepatology Communications*.

[10] Sterling R. K., Oakes T., Gal T. S., Stevens M. P., deWit M., Sanyal A. J. (2020). The fibrosis-4 index is associated with need for mechanical ventilation and 30-day mortality in patients admitted with coronavirus disease 2019. *The Journal of Infectious Diseases*.

[11] Pranata R., Yonas E., Huang I., Lim M. A., Nasution S. A., Kuswardhani R. A. T. (2021). Fibrosis-4 index and mortality in coronavirus disease 2019: a meta-analysis. *European Journal of Gastroenterology and Hepatology*.

[12] Xiang F., Sun J., Chen P.-H. (2021). Early elevation of fibrosis-4 liver fibrosis score is associated with adverse outcomes among patients with coronavirus disease 2019. *Clinical Infectious Diseases*.

[13] Ibáñez-Samaniego L., Bighelli F., Usón C. (2020). Elevation of liver fibrosis index FIB-4 is associated with poor clinical outcomes in patients with COVID-19. *The Journal of Infectious Diseases*.

[14] Sterling R. K., Lissen E., Clumeck N., Sola R., Correa M. C., Montaner J. (2021). Development of a simple non-invasive index to predict significant fibrosis in patients with HIV/HCV coinfection. *Hepatology*.

[15] Baik M., Nam H. S., Heo J. H. (2020). Advanced liver fibrosis predicts unfavorable long-term prognosis in first-ever ischemic stroke or transient ischemic attack. *Cerebrovascular Diseases*.

[16] Shah A. G., Lydecker A., Murray K. (2009). Comparison of noninvasive markers of fibrosis in patients with nonalcoholic fatty liver disease. *Clinical Gastroenterology and Hepatology*.

[17] Ginès P., Graupera I., Lammert F. (2016). Screening for liver fibrosis in the general population: a call for action. *The Lancet. Gastroenterology and hepatology*.

[18] Calapod O. P., Marin A. M., Onisai M., Tribus L. C., Pop C. S., Fierbinteanu-Braticevici C. (2021). The impact of increased fib-4 score in patients with type II diabetes mellitus on covid-19 disease prognosis. *Medicina*.

[19] Barron E., Bakhai C., Kar P. (2020). Associations of type 1 and type 2 diabetes with COVID-19-related mortality in England: a whole-population study. *Lancet Diabetes and Endocrinology*.

[20] Campos-Murguía A., Román-Calleja B. M., Toledo-Coronado I. V. (2021). Liver fibrosis in patients with metabolic associated fatty liver disease is a risk factor for adverse outcomes in COVID-19. *Digestive and Liver Disease*.

[21] Targher G., Mantovani A., Byrne C. D. (2020). Risk of severe illness from COVID-19 in patients with metabolic dysfunction-associated fatty liver disease and increased fibrosis scores. *Gut*.

[22] Vandenbroucke J. P., Von Elm E., Altman D. G. (2014). Strengthening the reporting of observational studies in epidemiology (STROBE): explanation and elaboration. *International Journal of Surgery*.

[23] Chen Z., Zhang F., Hu W. (2021). Laboratory markers associated with COVID-19 progression in patients with or without comorbidity: a retrospective study. *Journal of Clinical Laboratory Analysis*.

[24] McPherson S., Hardy T., Dufour J.-F. (2017). Age as a confounding factor for the accurate non-invasive diagnosis of advanced NAFLD fibrosis. *American Journal of Gastroenterology*.

[25] Wu Y., Li H., Guo X. (2020). Incidence, risk factors, and prognosis of abnormal liver biochemical tests in COVID-19 patients: a systematic review and meta-analysis. *Hepatology International*.

[26] Bloom P. P., Meyerowitz E. A., Reinus Z. (2021). Liver biochemistries in hospitalized patients with COVID-19. *Hepatology*.

[27] Lv X. H., Yang J. L., Deng K. (2021). Letter to the editor: COVID-19-Related liver injury: the interpretation for aspartate aminotransferase needs to be cautious. *Hepatology*.

[28] Crisan D., Avram L., Grapa C. (2021). Liver injury and elevated FIB-4 define a high-risk group in patients with COVID-19. *Journal of Clinical Medicine*.

[29] Park J. G., Kang M. K., Lee Y. R. (2020). Fibrosis-4 index as a predictor for mortality in hospitalized patients with COVID-19: a retrospective multicentre cohort study. *BMJ Open*.

[30] Saito Y., Okumura Y., Nagashima K. (2020). Impact of the fibrosis-4 index on risk stratification of cardiovascular events and mortality in patients with atrial fibrillation: findings from a Japanese multicenter registry. *Journal of Clinical Medicine*.

[31] Baik M., Kim S. U., Kang S. (2019). Liver fibrosis, not steatosis, associates with long-term outcomes in ischaemic stroke patients. *Cerebrovascular Diseases*.

[32] July J., Pranata R. (2021). Prevalence of dementia and its impact on mortality in patients with coronavirus disease 2019: a systematic review and meta-analysis. *Geriatrics and Gerontology International*.

[33] Peckham H., De Gruijter N. M., Raine C. (2020). Male sex identified by global COVID-19 meta-analysis as a risk factor for death and ITU admission. *Nature Communications*.

